# Effective removal of dyes from aqueous systems by waste-derived carbon adsorbent: physicochemical characterization and adsorption studies

**DOI:** 10.1038/s41598-025-13685-x

**Published:** 2025-08-06

**Authors:** Ali Ender Kuyucu, Ahmet Selçuk, Yunus Önal, İhsan Alacabey, Kadir Erol

**Affiliations:** 1 Department of Chemistry, Institute of Science, Yüzüncü Yıl University, Van, 65080 Turkey; 2 Faculty of Education, Department of Mathematics and Science Education, Yüzüncü Yıl University, Van, 65080 Turkey; 3 Faculty of Engineering, Department of Chemical Engineering, Inonu University, Malatya, 44280 Turkey; 4 Department of Medical Services and Techniques, Vocational School of Health Services, Artuklu University, Mardin, 47100 Turkey; 5 Department of Medical Services and Techniques, Vocational School of Health Services, Hitit University, Çorum, 19030 Turkey

**Keywords:** Activated carbon, Adsorption, Dye, Isotherm, Kinetic, Walnut shell, Chemistry, Materials science, Environmental chemistry

## Abstract

Due to their cost-effectiveness and high surface area, activated carbons are commonly used for the adsorption of dyes from aqueous solutions. In this study, activated carbon was synthesized from walnut shell waste via KOH activation (1:3 ratio), yielding a surface area of 2347.4 m²/g. Reactive Blue 19 and Reactive Red 195 adsorption behavior were studied under varying experimental conditions. These included natural pH values (6.8–7.2), dye concentrations between 50 and 1250 mg L⁻¹, and adsorbent dosages ranging from 0.1 to 1.0 g. Adsorption equilibrium was achieved within 150 min. The maximum adsorption capacities were found to be 1227.17 mg g⁻¹ for RB 19 and 235.74 mg g⁻¹ for RR 195. Isotherm modeling was conducted using Langmuir, Freundlich, Temkin, and Dubinin–Radushkevich models, with Freundlich providing the best fit for both dyes, indicating multilayer adsorption on heterogeneous surfaces. Thermodynamic analysis revealed that the adsorption processes were spontaneous and endothermic, with negative Gibbs free energy (*ΔG°*), positive enthalpy (*ΔH°*), and positive entropy (*ΔS°*) values. These results highlight the high adsorption performance and practical potential of walnut shell-derived activated carbon for dye removal from wastewater.

**Supplementary Information:**

The online version contains supplementary material available at 10.1038/s41598-025-13685-x.

## Introduction

Although humanity’s quality of life has increased with technology and industrialization in recent years, this development has brought many problems^1,2^. The most important of these problems is environmental pollution, which scientists are dealing with and will continue to deal with as long as humanity exists^3,4^. Solid, liquid, and gaseous pollutants from various pollutant sources harm the air, water, soil, and ecosystem. The release of large amounts of pollutants into the environment due to the rapid increase in industrial and agricultural activities globally remains a key challenge^5,6^. Pigments are a type of pollutant that must be extricated from wastewater before being released into the natural habitat, as they lead to toxicity and adverse effects on photosynthetic activity^7,8^.

The textile industry’s use of reactive pigments is highly productive^9,10^. Reactive pigments are azoic pigments used in painting cellulose fibers^11^. Over 10.000 tons of pigments are utilized commercially in the textile industry every year, and their disintegration is difficult due to their complex aromatic molecular structures and synthetic roots^12^. For this reason, the separation of pigments from wastewater has become important for the environment^13^. Pigments are extracted from wastewater using ozonation^14^, flocculation/coagulation^15^, photocatalysis^16^, reverse osmosis^17^, etc. Scientists have turned to the less hazardous adsorption technique since these technologies are expensive and dependent on chemicals. The adsorption process is an acceptable, effective, and, most importantly, environmentally friendly method for removing pigments and contaminants from wastewater^18^. Besides, the adsorption method has much potential for decontamination because of its adaptability, versatility, and extensive range of sorbents^19,20^. The analyte’s properties, the adsorbent type, and the wastewater matrix’s makeup significantly impact the adsorption technique’s effectiveness^21,22^.

Because of its large pore volume, activated carbon is a commonly used adsorbent in the adsorption process that may separate contaminants^23,24^. Activated carbon is of interest due to its adsorption capability and ease of manufacture^25,26^. Numerous investigations have been carried out to create an inexpensive process for creating activated carbon with a greater adsorption capacity^27,28^. Utilizing waste biomasses, which are abundant in nature, has grown in popularity to lower the cost of producing activated carbon^29^. Because biomass is carbon-rich, renewable, and primarily agricultural waste, it lowers manufacturing costs and facilitates the creation of high-quality activated carbons^30,31^. Fruit shells, wood, and timber waste are examples of raw materials used as biomass since they are easily obtained and inexpensive^32,33^.

In this study, activated carbon was synthesized from walnut shell waste (WHAC) through chemical activation using potassium hydroxide, and its dye adsorption performance was systematically investigated. The effects of initial dye concentration, contact time, and temperature on Reactive Red 195 (RR 195) adsorption and Reactive Blue 19 (RB 19) were examined. Furthermore, adsorption isotherms, kinetic models, and thermodynamic parameters were evaluated to elucidate the adsorption mechanism. While numerous previous studies have explored dye removal using various synthetic and biomass-derived adsorbents, this study introduces a cost-effective, highly porous activated carbon prepared via a straightforward KOH activation method. The resulting material exhibited an exceptionally high surface area (2347.40 m²/g) and dominant mesoporosity, leading to an unprecedented maximum adsorption capacity exceeding 1200 mg g⁻¹ for RB 19. This is the first report demonstrating a high adsorption capacity for RB 19 using WHAC. The simplicity of the synthesis process, combined with the material’s high efficiency and scalability, highlights this work’s novelty and practical applicability for industrial wastewater treatment.

## Materials and methods

### Materials

Fresh walnut shell wastes were collected from the gardens in Ağartı Village of Tuşba District of Van Province. Reactive Blue 19 (powder), potassium hydroxide (KOH, reagent grade, 90%, flakes), and hydrochloric acid (HCI, 37%, ACS reagent) were bought from Sigma-Aldrich Company (Hamburg, Germany). Reactive Red 195 (powder) was provided by Biosynth GmbH Company (Berlin, Germany). Activated carbon, which is used as an adsorbent in adsorption studies, was synthesized in the laboratory. The conductivity of pure water utilized in adsorption research is 18.2 MΩ.cm, and all other chemicals are of analytical quality.

### Preparation of raw material

Fresh walnut outer shell waste was used as raw material to prepare activated carbon for the experiments. The fresh walnut outer shells were left to dry at room temperature in a laboratory environment. The raw materials dried for approximately 10 days in a laboratory environment were filled into 10-liter plastic containers, and their mouths were closed with parafilm. Then, 10 g of these samples were taken and kept in an oven at 105 °C for 24 h to determine their moisture content. It was measured that there was 8.4% moisture in the walnut outer shell. The walnut shell provided was placed in steel tubs for carbonization in steel reactors and subjected to heat treatment employing a three-zone cylinder furnace. This heat treatment was carried out at 500 °C. The inert atmosphere in the system was provided with nitrogen gas, and the gas flow rate was set at 100 mL/min. The furnace was heated to the final temperatures with a heating rate of 5 °C/min and cooled again after staying at these temperatures for 1 h.

### Activation processes and activated carbon syntheses

Following carbonization, 10 g of walnut outer shells were weighed using a precision scale and chemically activated by mixing with KOH at a 1:3 weight ratio, which was selected based on both literature precedent^34,35^ and preliminary optimization studies conducted during the material preparation phase. The mixture was then dried in an oven at 105 °C for 24 h. After this process, it was completed with a heating rate of 10 °C/min in a tubular reactor and a holding period of 60 min at 800 °C in a nitrogen environment, and then cooling in nitrogen gas. After activation, 0.5 N HCl was added to the activated sample and boiled and washed several times with hot deionized water from a pure water device. After the grinding process, KOH-treated activated carbon was separated by waiting in a desiccator to be used in experimental studies.

The activation yield and burn-off percentage were calculated to assess the efficiency of the activation process and material loss. The activation yield was determined as the ratio of the final weight of activated carbon to the initial weight of the walnut shell precursor after carbonization and chemical activation. The burn-off percentage was calculated based on the mass loss during activation. The activation yield was 27.6%, while the burn-off percentage was calculated as 72.4%, indicating substantial removal of volatile components and efficient pore development. These values are consistent with chemically activated biomass-derived carbons in the literature and confirm the effectiveness of the KOH activation process.

### Adsorption studies

All adsorption studies were conducted in a batch system at the natural pH environment of dye RB 19 and RR 195 solutions. Preliminary adsorption trials were conducted at different pH values (ranging from pH 3.0 to 9.0) for both RB 19 and RR 195 dyes to evaluate the influence of pH on adsorption capacity. However, no significant variation in adsorption performance was observed across this pH range. To maintain experimental simplicity and better simulate real-world conditions, the adsorption experiments were performed at the natural pH values of the dye solutions: 6.8 for RB 19 and 7.2 for RR 195, without further adjustment. First, 1000 mg L^− 1^ stock solutions were prepared in 1 L volumetric flasks. The dye solutions were prepared 100 mL of 50, 100, 150, 200, and 250 mg L^− 1^ in 100 mL erlenmeyer flasks. Then, 0.1 g of activated carbon finely ground in an agate mortar was added to these flasks, and the solution was left to stir for 1 h with the help of a magnetic stirrer. To prevent possible evaporation during this process, the mouth of each erlenmeyer flask was tightly closed with parafilm. After 1 h, the stirring process was stopped, and samples were taken from each erlenmeyer flask, filtered with a membrane filter, and placed in cuvettes. Measurements were taken with a UV-VIS spectrophotometer (Shimadzu UV-2100 S, Japan).

The results were calculated using the following equation:

1 $$\:q=\:\frac{\left(Ci-Cf\right)\:x\:V}{m}$$

Here; *q* = Adsorbed dye amount (mg g^− 1^), *C*_*i*_ = Initial dye concentration (mg L^− 1^), *C*_*f*_= Measured dye concentration (mg L^− 1^), *m* = Activated carbon amount (g), *V* = Solution volume (L).

Table 1 summarizes key chemical and toxicological properties of RB 19 and RR 195 that influence their adsorption performance. UV-VIS spectrophotometric monitoring was performed before and after adsorption to verify that dyes were removed via adsorption rather than chemical degradation. The characteristic absorption peaks of RB 19 (593 nm) and RR 195 (540 nm) remained unchanged regarding wavelength position, indicating that the dye molecules retained their structural integrity. This confirms that the removal mechanism is dominated by physical adsorption rather than molecular degradation.

**Table 1:** Key physicochemical and toxicological properties of reactive dyes RB 19 and RR 195.

Property	RB 19	RR 195
Chemical formula	C₂₂H₁₆N₂Na₂O₁₁S₃	C₂₆H₁₈ClN₇Na₄O₁₉S₆
Molar mass (g/mol)	626.5	1120.1
Structure	Anthraquinone-based azo dye	Azo-based bifunctional dye
Solubility in water	Very soluble	Soluble
λ_max_ (nm)	~ 593	~ 540
pKa	~ 3.5–4.5 (sulfonic acid groups)	~ 4.0–5.0 (sulfonic and carboxylic groups)
LD₅₀ (oral, rat)	~ 5000 mg/kg (estimated, low toxicity)	~ 2000–3000 mg/kg (estimated)
Functional groups	–SO₃Na, –OH, –NH₂	–SO₃Na, –NH₂, –Cl, triazine ring
Dye class	Reactive anthraquinone	Reactive azo dye
Applications	Cotton dyeing, textile industry	Cellulose and protein fibers
Environmental impact	Persistent, resistant to biodegradation	Moderate persistence, complex degradation

### Modeling adsorption kinetics

The kinetics of the adsorption process were observed using pseudo-first-order, pseudo-second-order, Elovich, and intraparticle diffusion kinetic models. This study used a kinetic analysis approach for the adsorption process. We could derive important adsorption parameters from the experimental results using an appropriate kinetic model. These consist of the equilibrium adsorption capacity, rate constants, and starting adsorption rate.

The pseudo-first-order adsorption rate constant, expressed in min^− 1^, is denoted by *k*_*1*_. At equilibrium and time *t* (measured in minutes), the dye’s adsorption capacities are represented by the letters *q*_*e*_ and *q*_*t*_, respectively. The kinetic parameters were ascertained by analyzing the slope and y-intercept of linear graphs involving the natural logarithm of (*q*_*e*_ *- q*_*t*_)^36^.

2 $$\:\text{log}\left({q}_{e}-\:{q}_{t}\right)=\:\text{log}{q}_{e}-\:\left(\frac{{k}_{1}}{2.303}\right)t$$

The value denoted as *k*_*2*_ represents the pseudo-second-order adsorption rate constant (g mg^− 1^ min^− 1^). The adsorption capacities were determined in mg g^− 1^ at equilibrium (*q*_*e*_) and time *t* (*q*_*t*_), which is measured in minutes. To get the model constant, we utilized the slope and y-intercept of the pseudo-linear graph showing *t/q*_*t*_ against time (*t*)^36^.

3 $$\:\frac{t}{{q}_{t}}=\:\frac{1}{{k}_{2}{{q}_{e}}^{2}}+\:\frac{1}{{q}_{e}}t$$

Equation 4 represented the linear version of the Elovich kinetic model.

4 $$\:{q}_{t}=\:\frac{\text{ln}\alpha\:\beta\:}{\beta\:}+\:\frac{1}{\beta\:}\text{ln}t$$

*q*_*t*_ describes the adsorption capacity at time (*t*), and the slope and y-intercept of the graphical representation of *q*_*t*_ vs. In(*t*) were used to compute the parameters *α* (mg g^− 1^ min^− 1^), and *β* (g mg^− 1^)^36^.

Furthermore, the following is a representation of the intraparticle diffusion kinetic equation:

5 $$\:{q}_{t}=\:{k}_{i}{t}^{1/2}+C$$

Here, *k*_*i*_ is the intraparticle diffusion rate constant (mg g^− 1^ min^− 1/2^), and *C* is a constant (mg g^− 1^) that gives information about the thickness of the layer formed between the adsorbent and the adsorbate. The rate constant *k*_*i*_ is calculated from the slope of the graph of *q*_*t*_ against *t* ^*1/2*^. *C* is equal to the intercept value^37^.

### Adsorption isotherms

Langmuir, Freundlich, Temkin, and Dubinin–Radushkevich (D–R) isotherms have been examined to evaluate pollutant adsorption capacity to identify and comprehend maximum adsorption mechanisms. These are frequently employed to elucidate the nuances of the adsorption process^36^.

Initially created for gas-solid interactions, the Langmuir isotherm model has been used for various sorbents. According to this kinetics-based experimental model, there is neither net adsorption nor desorption at the surface at equilibrium. The following is a description of the Langmuir isotherm:

6 $$\:\frac{1}{{Q}_{eq}}=\:\frac{1}{{Q}_{max}b}\frac{1}{{C}_{eq}}+\:\frac{1}{{Q}_{max}}$$

The report displays *C*_*eq*_ (mg L^− 1^) as the equilibrium dye concentration in solution, *Q*_*eq*_ (mg g^− 1^) as the equilibrium unit’s adsorption capacity, *Q*_*max*_ (mg g^− 1^) as the adsorption capacity of an entire monolayer, and *b* (L mg^− 1^) as the constant denoted by the adsorption energy and binding site affinity^38^.

The Freundlich isotherm describes equilibrium on heterogeneous surfaces and does not assume a monolayer capacity since the amount of adsorbed substance increases with concentration in solution.

7 $$\:\text{ln}{Q}_{eq}=\:\frac{1}{n}\text{ln}{C}_{eq}+\:\text{ln}{K}_{F}$$

Here, *K*_*F*_ (L g^− 1^) and *n* (dimensionless) are Freundlich constants, adsorbent capacity, and heterogeneity factor, respectively. The slope of the line obtained from the graph drawn between *lnC*_*eq*_ and *lnQ*_*eq*_ gives 1/*n*, and the point where it intersects the ordinate gives *lnK*_*F*_^39^.

The Temkin adsorption isotherm expresses the indirect effects of adsorbate-adsorbate interactions on adsorption. According to this isotherm, the heat of adsorption of all molecules in the layer decreases linearly.

8 $$\:{Q}_{eq}=Bln{K}_{T}+\:Bln{C}_{eq}$$

Here, *B* is written instead of *RT/b* (*b*: Temkin isotherm constant, L mg^− 1^) and is found from the constants *A* and *B* and the graph of *lnC*_*eq*_ versus *Q*_*eq*_^40^. In the equation, *R* is the universal gas constant (8.314 J mol^− 1^ K^− 1^) and T is the environment temperature (K).

The D–R adsorption isotherm is an adsorption isotherm proposed for analysis in systems that give highly rectangular isotherms.

9 $$\:ln{Q}_{eq}=\:ln{Q}_{m}-{k}_{DR}{\epsilon\:}^{2}$$

In this equation, *Q*_*m*_ (mg g^− 1^) is the D–R monolayer capacity, *k*_*DR*_ (mg^2^ J^− 2^) is the constant related to the adsorption energy, and *ε* is the Poloni potential related to the equilibrium concentration, *R* is the universal gas constant (8.314 J mol^− 1^ K^− 1^), *T* is the temperature (K). *Q*_*m*_ and *k*_*DR*_ can be calculated from the *ε*^*2*^ versus *lnQ*_*eq*_ graph. The *k*_*DR*_ constant helps us find the adsorption energy (*E*). The adsorption energy indicates whether the adsorption mechanism is physical or chemical in character. The *E* value can be calculated with the following equation^40^.

10 $$\:E=({2{k}_{DR})}^{-1/2}$$

If the value of *E* is between 8 and 16 kJ mol^− 1^, the adsorption mechanism is chemical ion exchange. If the value of *E* is less than 8 kJ mol⁻¹, the process is considered physical in nature^40^.

## Results and discussion

### Characterization studies

Synthesized activated carbon and raw material were prepared for analysis by thoroughly grinding in an agate mortar. The ground-activated carbon was subjected to scanning electron microscopy (SEM, Leo EV040), X-ray diffractometry (XRD, Rigaku RadB-DMAX II), Brunauer-Emmett-Teller surface area (BET, Micromeritics Tristar 3000), elemental analysis (Leco CHNS-932), and Fourier Transform Infrared Spectroscopy (FT-IR, IRTracer − 100) analyses.

**Fig. 1:**
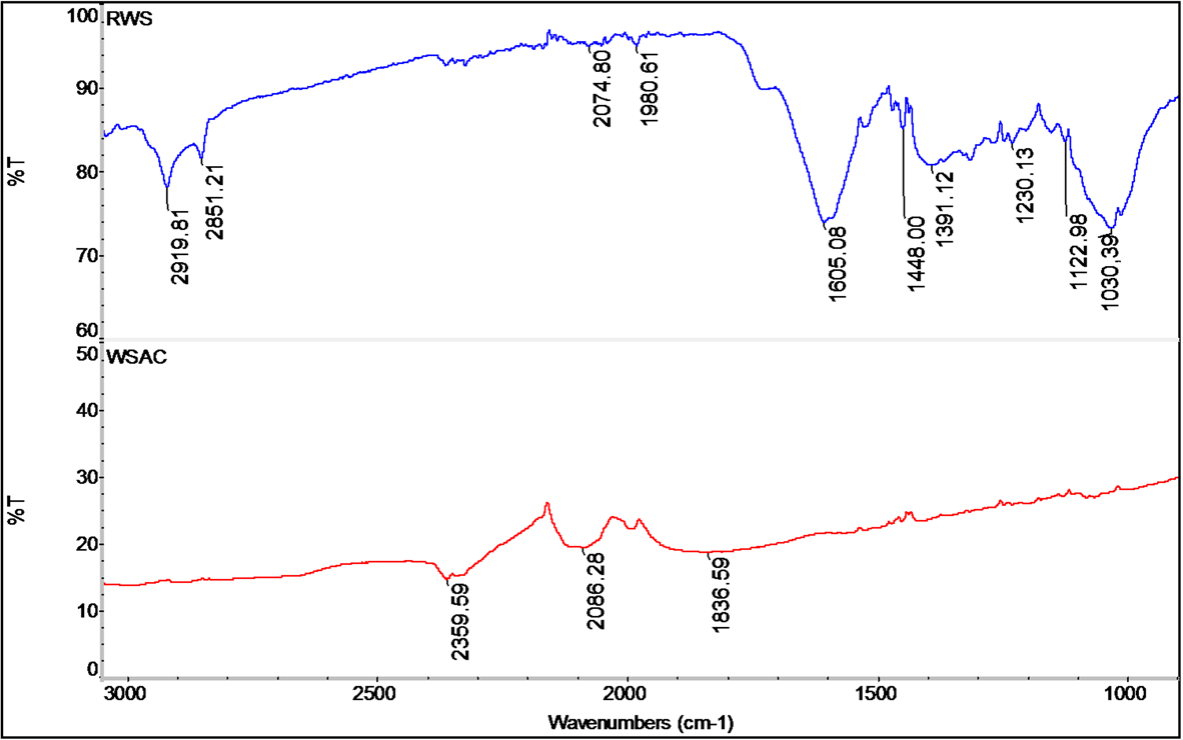
FTIR spectra of RWS and WSAC.

When the FTIR spectrum of the raw walnut shell (RWS) sample is examined, the flat peak seen around 3300 cm^− 1^ belongs to the stretches of –OH groups belonging to water and –OH groups in aromatic and inorganic structures (Fig. 1). The peaks around 2850 cm⁻¹ and 2919 cm⁻¹ are attributed to aliphatic C–H stretching vibrations, while the weak peak observed around 3000 cm⁻¹ corresponds to aromatic C–H stretches. The broad, sharp peak around 1605 cm^− 1^ is suitable for the stretches of the C = O group and the aromatic ring stretch in the lignin structure. In addition, the peak seen around 1030 cm^− 1^ belongs to organic C-O and C-O-C structures^41^.

Upon examining the FTIR spectrum of WSAC, the peak observed at 2359 cm^− 1^ is interpreted as originating from the muscular O = C = O stretch (Fig. 1). The peak at 2115 cm^− 1^ originates from the weak C ≡ C stretch of the alkyne group, and the peak observed at 1836 cm^− 1^ originates from the C-H stretching of aromatic compounds^42^.

**Fig. 2:**
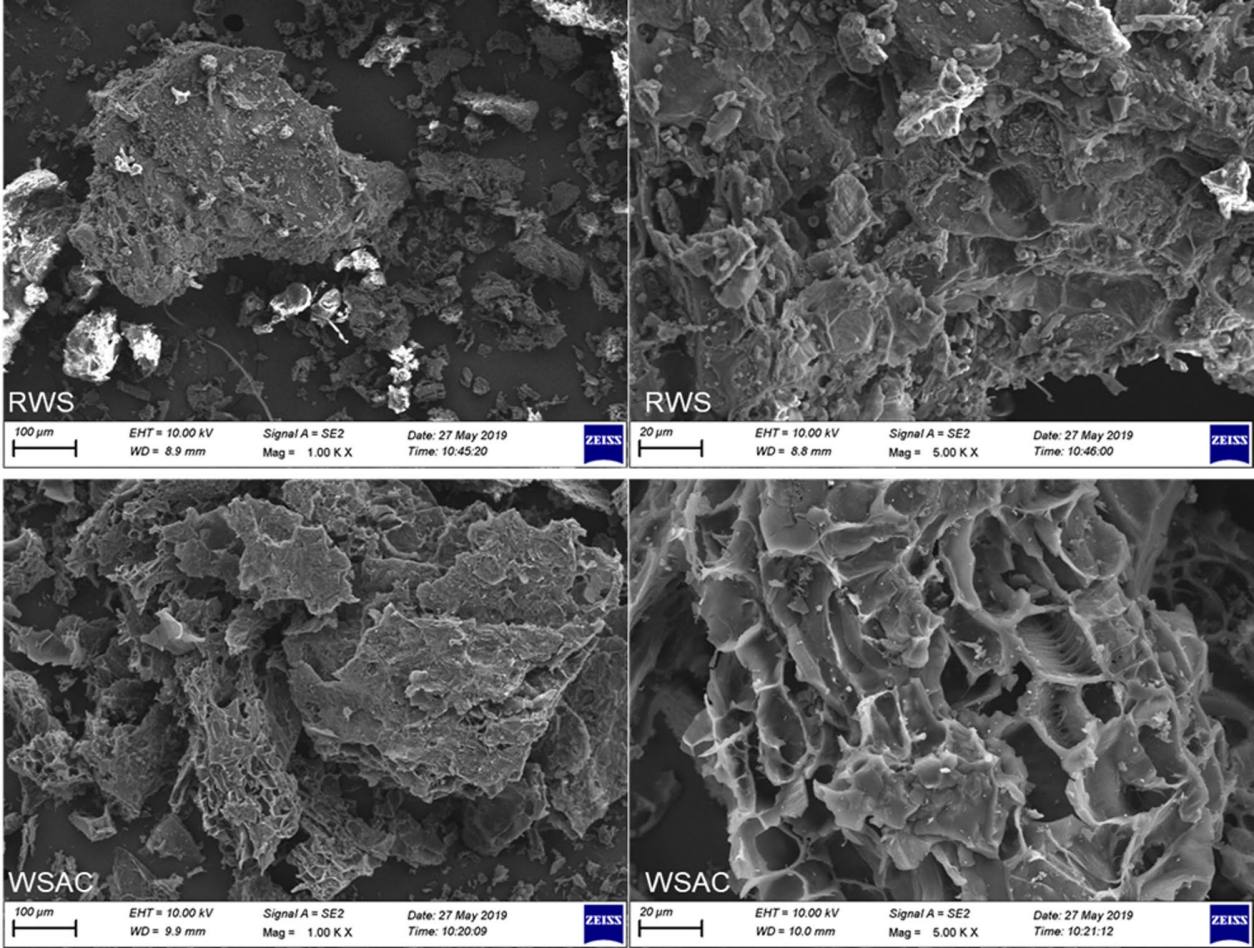
SEM images of RSW and WSAC.

Figure 2 shows the SEM micrographs of RWS and WSAC at different magnifications. In the RWS images (top row), a relatively dense and compact surface morphology is observed, with limited visible porosity and irregular, smooth textures. This structure reflects the natural, unactivated biomass matrix that hinders dye diffusion and adsorption. In contrast, the WSAC images (bottom row) reveal a highly porous and rough surface structure, significantly increasing surface fractures, cavities, and well-developed mesopores due to chemical activation with KOH. The high-magnification image clearly illustrates the formation of an extensive porous network, including interconnected channels and voids, which facilitate enhanced mass transfer and adsorptive interactions. This morphological transformation confirms the successful activation and explains the substantial improvement in adsorption performance observed in subsequent dye removal experiments.

Moreover, BET device measurements created the values ​​given in Table 2. In the measurements, the area covered by WSAC micropores was 18.20 m^2^/g, while the area covered by mesopores was 2329.19 m^2^/g. The fact that more mesopores are needed for WSAC indicates that the surface area is large. The surface area measured in the BET device was 2347.40 m^2^/g.

**Table 2:** WSAC’s BET analysis results.

S_BET_ (m^2^/g)	S_micro_ (m^2^/g)	S_meso_ (m^2^/g)	V_T_ (cm^3^/g)	V_micro_ (cm^3^/g)	V_meso_ (m^3^/g)	dp (nm)
2347.40	18.20	2329.19	1.28	0.18	0.19	2.19

**Fig. 3:**
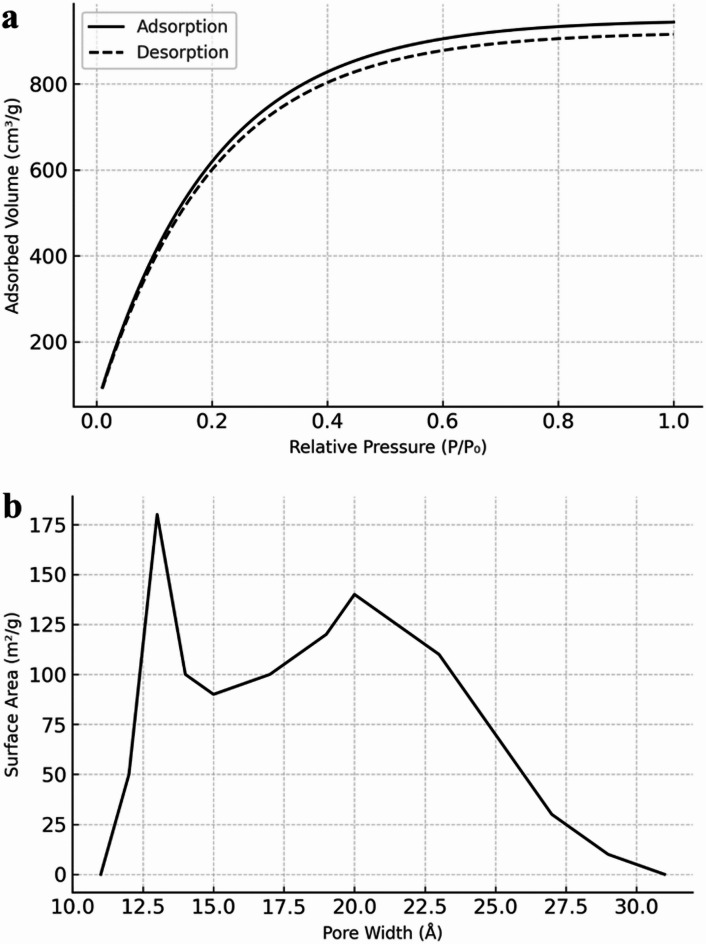
(**a**) Nitrogen adsorption–desorption isotherm of WSAC. (**b**) Pore size distribution of WSAC.

Figure 3a presents the nitrogen adsorption–desorption isotherm curve of WSAC. According to the IUPAC classification, the isotherm corresponds to Type IV with an H4 hysteresis loop, indicating the presence of mesoporous structures. This observation agrees with the BET analysis results summarized in Table 2, which reveal a dominant mesoporous surface area. Figure 3b, on the other hand, illustrates the pore size distribution curve of WSAC derived from BJH analysis. The distribution indicates that most of the pore volume lies within the mesoporous range (~ 15–30 Å), which further confirms the mesoporous nature of the material. The isotherm shape, pore size distribution, and textural parameters confirm that WSAC possesses a high surface area and well-developed mesoporosity, significantly enhancing its adsorption performance for dye molecules in aqueous systems.

**Fig. 4:**
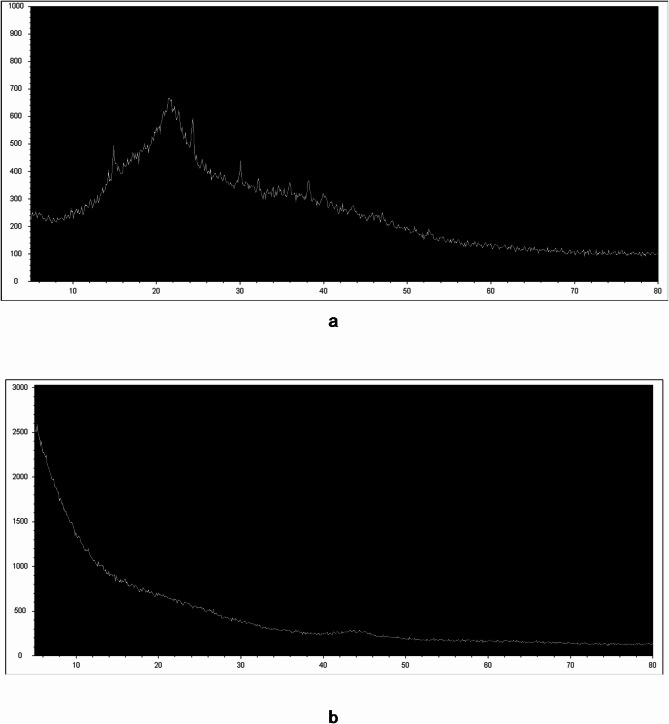
XRD spectrum of (**a**) RWS, (**b**) WSAC.

Figure 4 shows the XRD spectra of RWS and WSAC structures. When the results of RWS are examined, it is seen that the sample is mainly amorphous and also contains crystalline structures. A single homogeneous structure, such as the macromolecular structure, is semi-crystalline (Fig. 4a). In the spectrum of WSAC, it was noted that the synthesized activated carbon evolved into a completely amorphous structure in direct proportion to the increasing amount of KOH. There are three different amorphous structures in the macromolecular structure of activated carbon. Partially crystalline regions were also observed in the structure of activated carbon (Fig. 4b).

However, the elemental analysis results are presented in Table 3. When the SWAC sample was compared with the RWS, the amount of carbon in the synthesized activated carbon increased from 40.47 to 86.93%. These results indicate that biomass waste represents a promising raw material for activated carbon synthesis. In addition, the amount of oxygen in the raw sample decreased from 53.8 to 12.8%. This is a practical result of the activation mechanism with KOH.

**Table 3:** Elemental analysis results.

RWS		WSAC	
Element	Amount (%)	Element	Amount (%)
N	1.022	N	–
C	40.479	C	86.935
H	4.703	H	0.298
O	53.795	O	12.766

### Adsorption–desorption studies

**Fig. 5:**
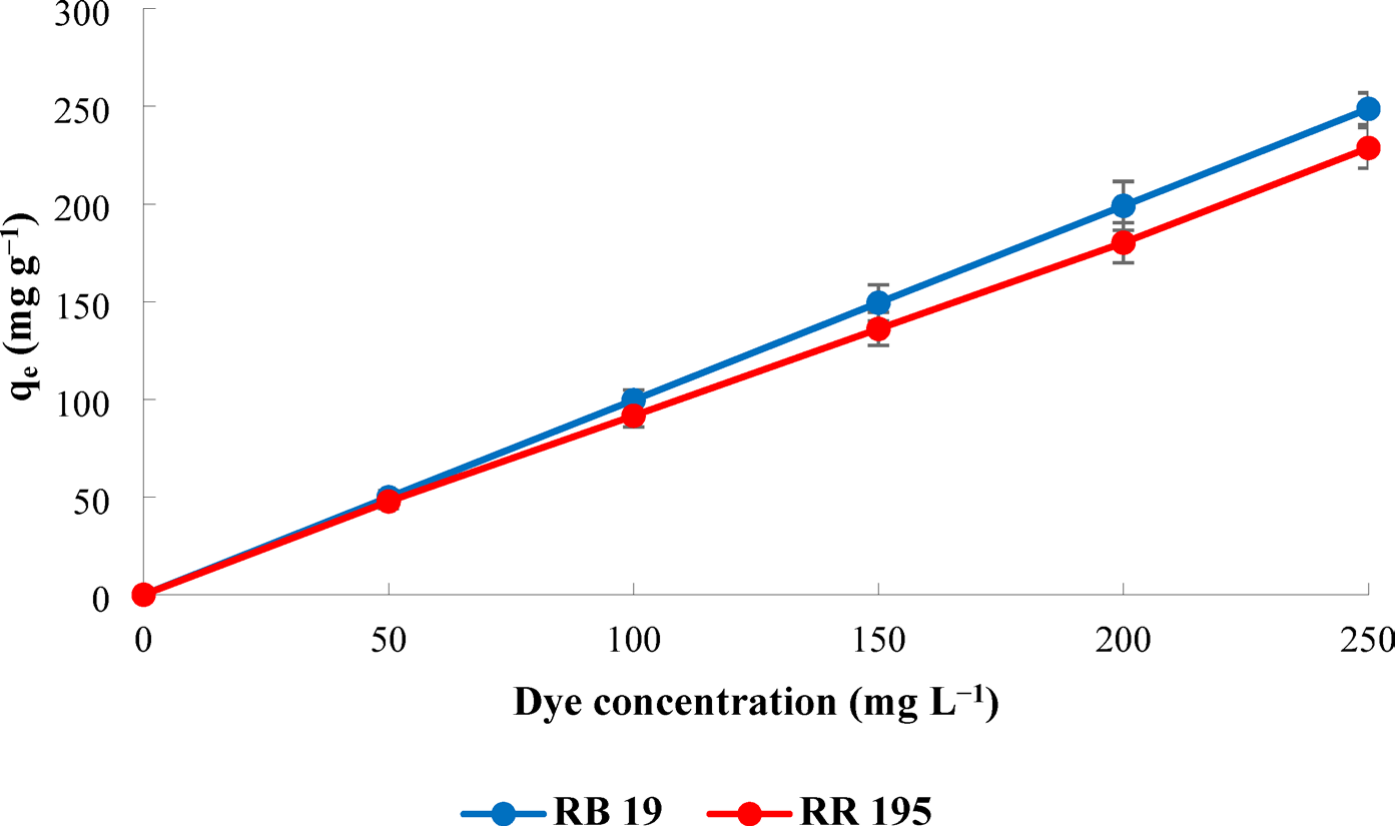
Effect of dye concentrations on adsorption capacities.

Within the scope of adsorption studies, firstly, to determine whether the adsorption of both dyes with WSAC was suitable, adsorption studies were carried out in the 50–250 mg L^− 1^ concentration range with 0.1 g of adsorbent and 100 mL of dye volume. 60 min was deemed sufficient as the interaction time. After filtration with the help of a membrane filter, studies were carried out to determine the concentrations at the specific wavelengths for both dyes in the UV-VIS device. The results obtained are presented graphically in Fig. 5. As can be seen, increasing dye concentrations also increased the adsorption capacities.

**Fig. 6:**
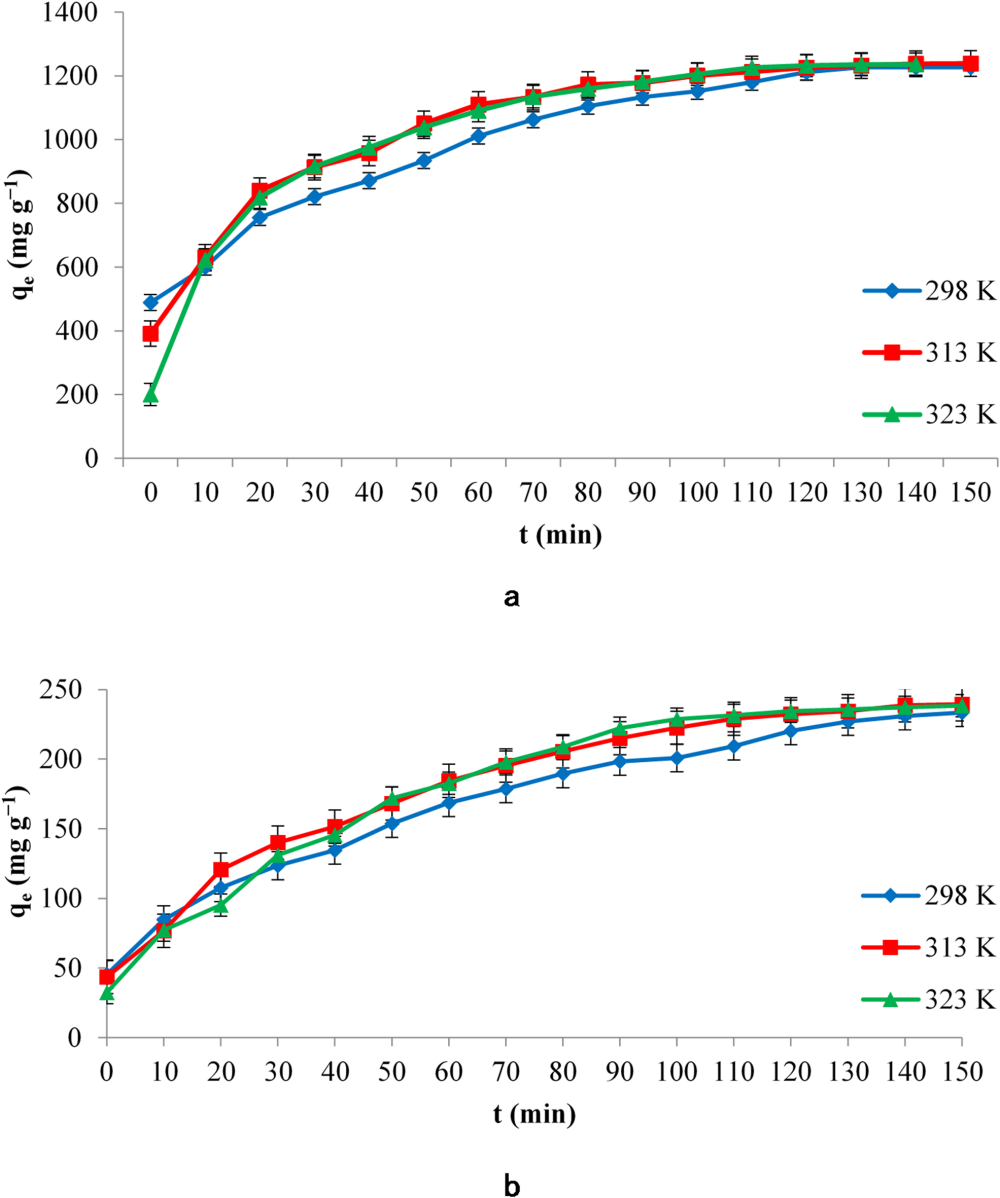
Effect of different temperatures and interaction times on (**a**) RB 19, (**b**) RR 195 adsorption.

After ensuring that both dyes were adsorbed with WSAC, adsorptions were carried out in the range of 0–150 min at high dye concentration (1250.0 mg L^− 1^), high adsorbent dose (1.0 g), and large solution volume (1 L). High dye concentration and adsorbent dose were selected for the extended adsorption experiments to ensure measurable and distinguishable adsorption profiles, especially for isotherm and kinetic modeling at high loading. The high initial dye concentration helps better evaluate the activated carbon’s maximum adsorption capacity and simulates conditions in industrial wastewater where dye levels can be significantly elevated. Similarly, the increased adsorbent dose ensures sufficient active sites to approach equilibrium within a reasonable interaction time and provides more accurate kinetic data. This study was conducted at three different temperatures: 25 °C, 40 °C, and 50 °C. The change graph of the amount of dye adsorbed by WSAC over time is given in Fig. 6.

When the graphs were examined, it was seen that the adsorption rate of the activated carbon for RB 19 increased with the increase in temperature, and the solution cleaning time decreased. For RR 195, the adsorption rate of the activated carbon increased relatively with the increase in temperature, and the solution cleaning time decreased similarly.

The initially high adsorption rate indicates that the activated carbon’s outer surface (film layer) is the first to be occupied. After filling the outer surface, the adsorbate ions move more slowly into the pores in the following periods. This movement can be considered as the diffusion of adsorbate molecules into the grains, which is the limiting step of the adsorption rate. The last step is the step where adsorption reaches equilibrium, and since saturation is reached at this stage, many fewer adsorbate molecules are adsorbed. After this stage, adsorption occurs very slowly^43^.

### Kinetic studies

When the adsorption data of RB 19 and RR 195 dyes with WSAC are examined, it is seen that both adsorption mechanisms comply with pseudo-second-order kinetics, where R^2^ values ​​are higher. When we consider the components of active carbon amount + dye concentration + solvent amount, since the solvent (water) concentration is very high, the adsorption kinetics should be pseudo-second order. The pseudo-second-order kinetic model’s applicability demonstrates no diffusion issues throughout the adsorption phase. The adsorption rate appears to be influenced by strong surface interactions, such as electrostatic attraction and π–π stacking, which are sufficient to yield second-order kinetics, even without an actual chemisorption mechanism. As stated in the article, the adsorption process of RB 19 and RR 195 with WSAC is controlled chemically^44^. In addition, the high R^2^ values ​​in the first-order kinetics graph can be explained by the high dye concentration next to the active carbon concentration.

In the Elovich model, in reactions involving the chemical adsorption of gases on a solid surface without desorption of products, the rate decreases with time due to the increase in surface coverage. In addition, the Elovich equation assumes that actual solid surfaces are energetically heterogeneous and that neither desorption nor interaction between adsorbed species can significantly affect the adsorption kinetics at low surface coverage. The critical effect of surface energy heterogeneity on the adsorption equilibrium has been demonstrated in gas/solid systems, but its extension to liquid/solid systems is unknown^45^. When Tables 4 and 5 are examined, it is seen that the adsorption of both dyes on activated carbon partially fits the Elovich kinetic model. Although the adsorption process is predominantly physical, as thermodynamic and isotherm analyses indicate, the pseudo-second-order kinetic model best fits the data. This suggests that the adsorption rate is governed by surface interactions and diffusion-controlled mechanisms rather than a truly chemical reaction.

In the intraparticle diffusion model, where adsorption occurs in three stages, the dye molecules move from the leading solution to the surface in the initial adsorption stage. The dye molecules reaching the surface not only form a boundary layer on the surface of the adsorbent but also settle in the pores and proceed to the later stages of adsorption. The adsorption process occurs more slowly at this stage. This is an expected event, and the molecules accumulating on the surface settle into the pores in a particular order. This stage corresponds to the second region of the intraparticle diffusion model, where dye molecules gradually diffuse into the pores of the adsorbent. The final stages of adsorption occur quite slowly, depending on the diameter and shape of the pores, whether open or closed at both ends, and many other factors. In this stage, depending on the progression of time, the adsorbate molecules fill the adsorbent pores, and adsorption slows down considerably.

When the boundary layer thicknesses of all three regions for both dyes in the model in question are examined, it can be said that the boundary layer thickness increases as the temperature increases in the first region, decreases for the second and third regions, and even remains constant.

When Tables 4 and 5 are examined, it is seen that the boundary layer thicknesses increase with temperature in all three stages of the model. It is seen that the increase in RB 19 dye is much more significant than the increase in RR 195 dye for both dyes. For example, for 25 °C temperature, the boundary layer thickness in the three regions is 56.006, 151.11, and 225.54 mg g⁻¹ for RR 195 dye, while it is 412.76, 920.44, and 1226.8 mg g⁻¹ for RB 19 dye. This situation is related to the dye-adsorbent interaction as well as the molecular size and physical and chemical structure of the dye. In fact, the molecular diameter of RR 195 dye is larger than the molecular diameter of RB 19 dye^46^.

**Table 4:** Kinetic parameters of the RB 19 adsorption mechanism.

	298 K			313 K			323 K		
Pseudo-first-order kinetic model									
*k*_*1*_ (min⁻¹)	0.079			0.44			0.51		
*q*_*e (calc)*_ (mg g⁻¹)	551.44			327.42			374.11		
*q*_*e (exp)*_ (mg g⁻¹)	1227.17			1238.92			1236.00		
R²	0.86			0.91			0.88		
Pseudo-second-order kinetic model									
*k*_*2*_ (g mg⁻¹ min⁻¹)	0.0002			0.0004			0.0004		
*q*_*e (cal)*_ (mg g⁻¹)	1250			1250			1250		
*q*_*e (exp)*_ (mg g⁻¹)	1227.17			1238.92			1236.00		
R²	0.99			0.99			0.99		
Elovich kinetic model									
$\:\beta\:$ (g mg⁻¹)	0.006			0.007			0.007		
*α* (mg g⁻¹ min⁻¹)	8413.02			15020.5			27641.18		
*q*_*e (exp)*_ (mg g⁻¹)	1227.17			1238.92			1236.00		
R²	0.95			0.86			0.94		
Intraparticle diffusion model									
Region	1	2	3	1	2	3	1	2	3
*k*_*i*_ (mg g⁻¹ min⁻^0.5^)	165.4	35.98	0.026	194.15	21.85	0.37	161.14	25.47	0.03
*C* (mg g⁻¹)	412.76	920.44	1226.8	401.98	1053.6	1234.5	516.7	1034.0	1235.7
R²	0.92	0.94	0.99	0.79	0.88	0.84	0.94	0.90	0.90

**Table 5:** Kinetic parameters of the RR 195 adsorption mechanism.

	298 K			313 K			323 K		
Pseudo-first-order kinetic model									
*k*_*1*_ (min⁻¹)	0.026			0.027			0.027		
*q*_*e (calc)*_ (mg g⁻¹)	95.61			97.70			86.94		
*q*_*e (exp)*_ (mg g⁻¹)	235.74			239.56			238.53		
R²	0.98			0.98			0.98		
Pseudo-second-order kinetic model									
*k*_*2*_ (g mg⁻¹ min⁻¹)	0.0007			0.0001			0.0001		
*q*_*e (cal)*_ (mg g⁻¹)	243.9			243.9			243.9		
*q*_*e (exp)*_ (mg g⁻¹)	235.74			239.56			238.53		
R²	0.99			0.99			0.99		
Elovich kinetic model									
$\:\beta\:$ (g mg⁻¹)	0.032			0.029			0.027		
*α* (mg g⁻¹ min⁻¹)	448.23			449.12			316.55		
*q*_*e (exp)*_ (mg g⁻¹)	235.74			239.56			238.53		
R²	0.97			0.96			0.93		
Intraparticle diffusion model									
Region	1	2	3	1	2	3	1	2	3
*k*_*i*_ (mg g⁻¹ min⁻^0.5^)	29.82	7.81	0.66	32.73	6.01	0.01	39.93	8.92	0.04
*C* (mg g⁻¹)	56.00	151.11	225.54	58.74	181.33	239.44	36.47	165.98	239.14
R²	0.99	0.94	0.97	0.95	0.86	0.93	0.96	0.80	0.99

In addition to the linear regression approach, non-linear kinetic modeling was carried out to evaluate the adsorption mechanism better. The model performance was assessed using error functions, including root mean square error (RMSE), sum of squared errors (SSE), and chi-square (χ²), which provide a quantitative measure of fit between experimental and model-predicted *q*_*e*_ values. Table 6 shows that the pseudo-second-order and intraparticle diffusion models exhibited the lowest error values for RB 19 and RR 195 dyes, confirming their strong agreement with experimental data. In contrast, the pseudo-first-order model resulted in significantly higher errors, indicating a poor fit. These findings support the conclusion that adsorption kinetics are best described by pseudo-second-order and intraparticle diffusion mechanisms, with contributions from surface interactions and pore diffusion.

**Table 6:** Non-linear kinetic model error metrics for RB 19 and RR 195 dyes.

Dye	Model	RMSE	SSE	Chi-square (χ²)
RB 19	Pseudo-first-order	822.66	2,030,298	1643.71
	Pseudo-second-order	16.73	840.0	0.68
	Elovich	0.00	0.00	0.00
	Intraparticle diffusion	2.57	19.76	0.016
RR 195	Pseudo-first-order	144.61	62740.20	263.64
	Pseudo-second-order	6.17	114.26	0.48
	Elovich	0.00	0.00	0.00
	Intraparticle diffusion	5.90	104.43	0.44

### Isotherm studies

Table 7 presents the parameters calculated from four different isotherm models. When both dyes are examined, the Freundlich model comes to the fore in adsorption. Accordingly, both dyes were adsorbed in multilayers on the adsorbent used in the study. Due to the content of active binding centers in different centers on heterogeneous surfaces, the Freundlich model is a more realistic approach compared to the Langmuir model. The correlation coefficient R^2^, having a high value such as 0.98 − 1.00, shows that the adsorbent tends to adsorb and has high adsorption capacity. Again, according to the Freundlich isotherm equation, for the heterogeneity factor *n*, if *n* = 1.0, adsorption is linear; if *n* < 1.0, adsorption is a chemical process, and if *n* > 1.0, adsorption occurs in a physical process^40^. However, a more nuanced interpretation suggests that *n* > 1.0 primarily reflects favorable adsorption, irrespective of the specific mechanism. When the *n* values in our study are examined, it is evident that both RB 19 and RR 195 dyes exhibit favorable adsorption onto WSAC, which is consistent with the high adsorption capacities obtained^38^.

Among the Temkin isotherm constants, *B* shows the binding energy when equilibrium is reached. The other constant, *K*_*T*_, is a variable that depends on the heat of adsorption. It was determined that the *B* value reached a higher value for RB 19 dye compared to RR 195 dye.

The microporosity of the adsorbent is the fundamental parameter in the application of the D–R model. The presence of both micro and mesopores in the active carbon subject to the study is a limiting parameter in the suitability of the examined dyes to the D–R model. Therefore, the adsorbent containing mesopores can explain the low R^2^ values ​​for both dyes. In addition, the relatively low R² values obtained from the D–R model indicate a weak correlation between the model and experimental data, thereby undermining the model’s reliability in describing the actual adsorption behavior. This inconsistency is likely attributed to the activated carbon’s micro- and mesoporous structures, which deviate from the D–R model’s fundamental assumption of micropore-limited adsorption. As the D–R model is primarily designed for systems dominated by microporous adsorption mechanisms, its applicability becomes limited in materials with significant mesoporosity and heterogeneous surface energy distributions, as observed in this study. The *E* value specified in Eq. 10 from the D–R isotherm relationship was calculated as 2.886 kJ mol^− 1^ for the adsorption of RB 19 dye and 0.707 kJ mol^− 1^ for the adsorption of RR 195 dye. In light of this information, it can be said that both adsorption processes are physical. These forces are more dominant than ion exchange and intraparticle diffusion. The adsorption isotherm plots used to evaluate the fitting of models are provided in the Supplementary Information (Figure S1-S4).

**Table 7:** Adsorption paramaters.

Dye	Langmuir			Freundlich			
	K_L_ (L mg^−1^)	q_m_ (mg g^−1^)	*R* ^2^	*n*	1/*n*	K_F_ (L g^−1^)	*R* ^2^
RB 19	0.22	1250.00	0.94	1.56	0.64	207.49	0.98
RR 195	0.05	370.37	0.65	1.52	0.66	25.97	0.96

Dye	Temkin			D–*R*			
	B (kJ mol^−1^)	K_T_ (L mg^−1^)	*R* ^2^	q_m_ (mg g^−1^)	E (kJ mol^−1^)	*R* ^2^	
RB 19	197.70	1.46	0.85	390.53	2.886	0.64	
RR 195	70.67	1.46	0.83	159.83	0.707	0.75	

### Thermodynamic studies

The thermodynamic data of the adsorption interaction of both dyes are presented in Table 8. When the results in the table are examined, it is seen that *ΔH°* is positive for both dyes. A positive *ΔH°* indicates that the adsorption process is endothermic, and a negative *ΔG°* value explains that the adsorption process occurs thermodynamically spontaneously. The decrease in *ΔG°* with increasing temperature indicates enhanced adsorption efficiency at elevated temperatures. A positive *ΔS°* value argues that the randomness in the solid/solution boundary layer increases, and the attraction power of the dye ions by the adsorbent and structural changes in the dye ions and the adsorbent. The effect of temperature on the adsorption process, including the van’t Hoff plots used for thermodynamic calculations, is shown in the Supplementary Information (Figure S5).

**Table 8:** Thermodynamic parameters.

	T (K)	lnK_c_^o^	ΔH^o^ (kJ mol^−1^)	ΔS^o^ (kJ mol^−1^ K^−1^)	ΔG^o^ (kJ mol^−1^)
RB 19	298	3.980	14.86	82.91	− 9.84
	313	4.239			− 11.09
	323	4.450			− 11.92
RR 195	298	2.496	24.54	103.48	− 6.30
	313	3.133			− 7.85
	323	3.234			− 8.88

### Comparison with past studies

Data related to some adsorption processes where the dyes used in our study were used before are given in Table 9. Accordingly, whether the adsorbent is in particle, film, or bulk structure, these two dyes are materials that have found a place in many studies. Removing these dyes from aqueous systems is an essential issue for environmental health, and studies have been carried out on many different adsorbents from the past to the present. When the adsorption capacity values ​​obtained with the results of these studies are compared with those ​​obtained in this study, it can be easily said that WSAC adsorbent is an effective adsorbent for RB 19 and RR 195 dyes.

The exceptionally high adsorption capacity of 1227.17 mg g^− 1^ obtained for RB 19 can be explained by several synergistic factors related to both the adsorbent characteristics and dye-specific properties. WSAC exhibited an extremely high specific surface area (2347.40 m²/g) and dominant mesoporosity, facilitating efficient diffusion and dye molecule adsorption. The smaller molecular size of RB 19 compared to RR 195 allowed for deeper penetration into these mesopores, enabling more effective utilization of the internal surface area. Furthermore, the aromatic structure of RB 19 promoted stronger π–π interactions with the oxygen-containing functional groups introduced during KOH activation, enhancing the adsorption affinity. The use of high initial dye concentrations increased the mass transfer driving force, thereby accelerating the adsorption process. These combined effects, surface accessibility, molecular compatibility, and favorable interaction mechanisms, account for the significantly higher uptake of RB 19 relative to RR 195. The close agreement between experimental and theoretical adsorption values obtained from pseudo-second-order kinetics, along with high correlation coefficients for the Freundlich and Langmuir isotherms, confirms the reliability of the data and underscores the superior performance of WSAC as an efficient adsorbent for RB 19 removal.

Unlike most studies summarized in Table 9, which often employ adsorbents with lower surface areas, limited porosity control, or require complex synthesis routes, our work introduces a cost-effective and scalable method for producing high-performance activated carbon from walnut shells with a remarkably high surface area and dominant mesoporosity. This is the first known study reporting an adsorption capacity exceeding 1200 mg g⁻¹ for RB 19 using walnut shell-derived activated carbon. The combination of a simple activation process, high adsorption capacity, and consistent performance across different dyes and models highlights the novelty and practical relevance of the present work.

**Table 9:** Adsorption results were obtained from some studies on RB 19 and RR 195.

Adsorbent	Dye	q_max_ (mg g^−1^)	Refs.
Chitosan-ZnO nanocomposite	RB 19	219.60	^47^
Chitosan films	RB 19	799.00	^48^
Chitosan-tripolyphosphate/MgO/Fe_3_O_4_	RB 19	120.30	^49^
Activated carbon coated ZnO nanoparticles	RB 19	94.33	^50^
Nano-sized MgO	RB 19	250.00	^51^
Lignin-based polyporous Carbon@polypyrrole	RB 19	537.52	^52^
TiO_2_ nanoparticles	RR 195	86.96	^53^
*Adenanthera Paronina* L seed activated carbon	RR 195	64.42	^54^
Chitosan/oxalic acid hydrogel	RR 195	110.70	^55^
Switchgrass biochar	RR 195	1288.40	^56^
Barberry stem powder	RR 195	27.20	^57^
Chitosan particles	RR 195	82.10	^58^
Walnut Shells Activated Carbon (WSAC)	RB 19	1227.17	This study
Walnut Shells Activated Carbon (WSAC)	RR 195	235.74	This study

It should be noted that some of the cited adsorption capacities, such as the 1288.4 mg g^− 1^ reported for switchgrass biochar, were obtained under highly optimized experimental conditions, including high initial dye concentrations and extended contact times. These values often represent maximum monolayer adsorption capacities derived from Langmuir isotherm modeling. They may not be directly comparable without considering normalization methods and test parameters such as pH, temperature, and adsorbent dose.

## Conclusion

Our study investigated the removal of dyes in wastewater by the adsorption method using activated carbon. Activated carbon with a 2347.40 m^2^/g surface area was obtained from the walnut shell by chemical activation with KOH. Using this activated carbon with WSAC code. Adsorption and kinetic studies were carried out for RB 19 and RR 195 dyes. In the adsorption studies, the adsorption capacity was reached as 1227.17 mg g^− 1^ for RB 19 and 235.74 mg g^− 1^ for RR 195 at 25 °C temperature and 150 min interaction time. Within the scope of kinetic modeling of the adsorption process, it was understood from the calculations that the adsorption mechanism for RB 19 and RR 195 dyes was more suitable for the pseudo-second-order kinetic model. In isotherm studies, it was determined from the calculations that both dye adsorptions were more suitable for the Freundlich adsorption isotherm. Finally, a thermodynamic investigation of the adsorption processes was carried out. The result of the positive *ΔH°* value indicated that both adsorption processes were endothermic. The negative values of *ΔG°* supported the spontaneity of adsorption processes. Also, the positive value of *ΔS°* showed that activated carbons have a high affinity for dyes. Given its high adsorption capacity, cost-effective preparation, and use of readily available biomass, the synthesized activated carbon shows strong potential for practical implementation in wastewater treatment plants, particularly for large-scale dye removal applications in the textile industry.

## Supplementary Information

Below is the link to the electronic supplementary material.

Supplementary Material 1

## Data Availability

The datasets used and/or analyzed during the current study are available from the corresponding author (Kadir Erol) upon reasonable request.
