# Supplementary material for: Effective removal of dyes from aqueous systems by waste-derived carbon adsorbent: physicochemical characterization and adsorption studies
Source: Sci Rep. 2025 Aug 6;15:28835. doi: 10.1038/s41598-025-13685-x (PMC12328673; doi:10.1038/s41598-025-13685-x)
Supplement: Supplementary file 1 — Supplementary Material 1 [file 41598_2025_13685_MOESM1_ESM.docx]

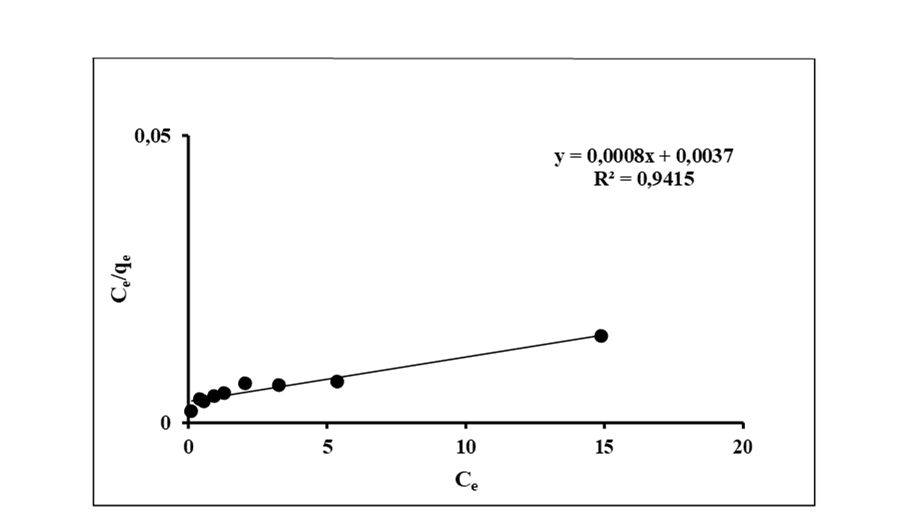


**a**

***
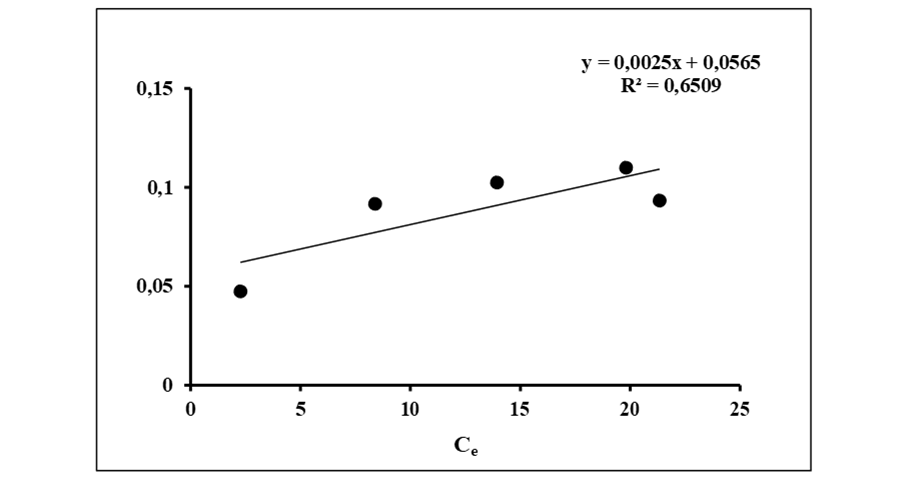
***

**b**

**Supplementary Figure S1.** Langmuir adsorption plot for a) RB 19, b) RR195 adsorption.


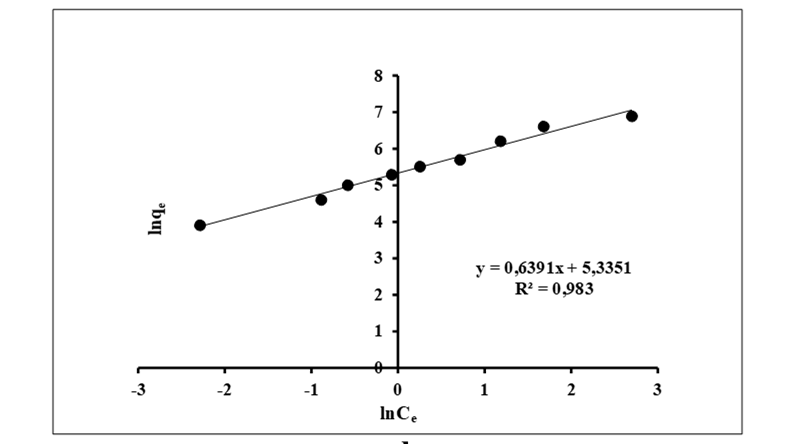


**a**


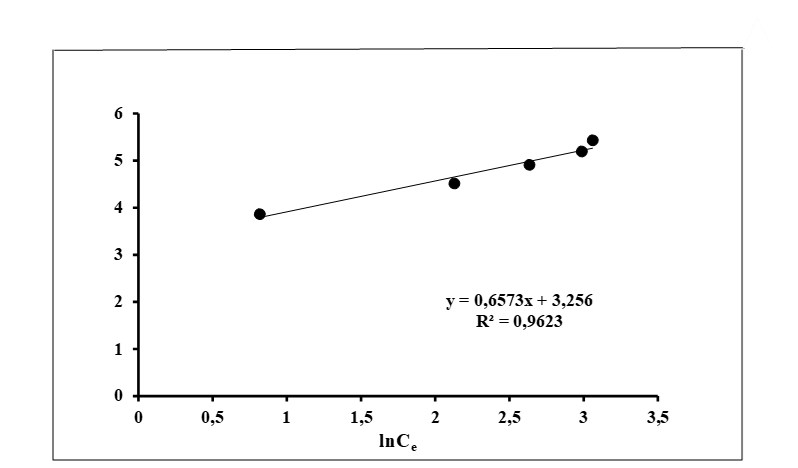


**b**

**Supplementary Figure S2.** Freundlich adsorption plot for a) RB 19, b) RR195 adsorption.

**
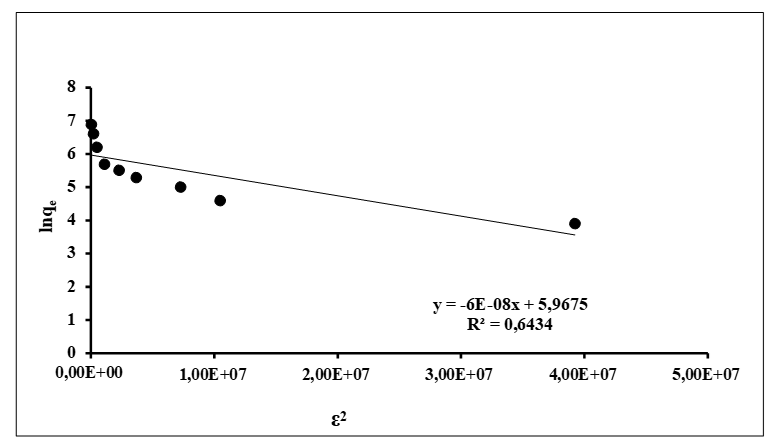
**

**a**

**
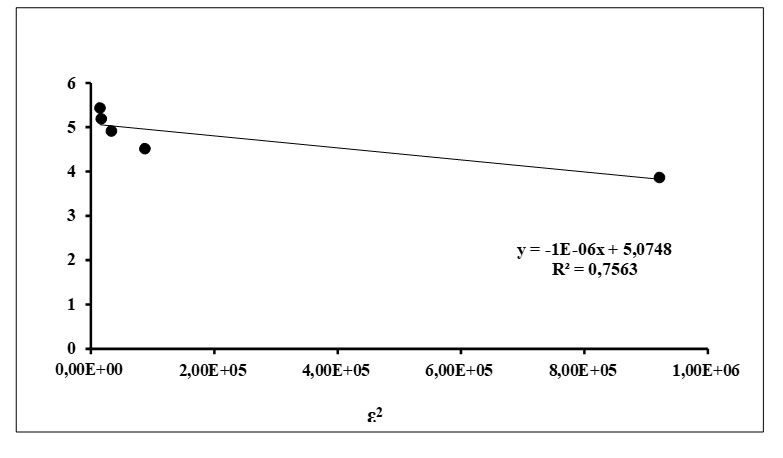
**

**b**

**Supplementary Figure S3.** D-R adsorption plot for a) RB 19, b) RR195 adsorption.

**
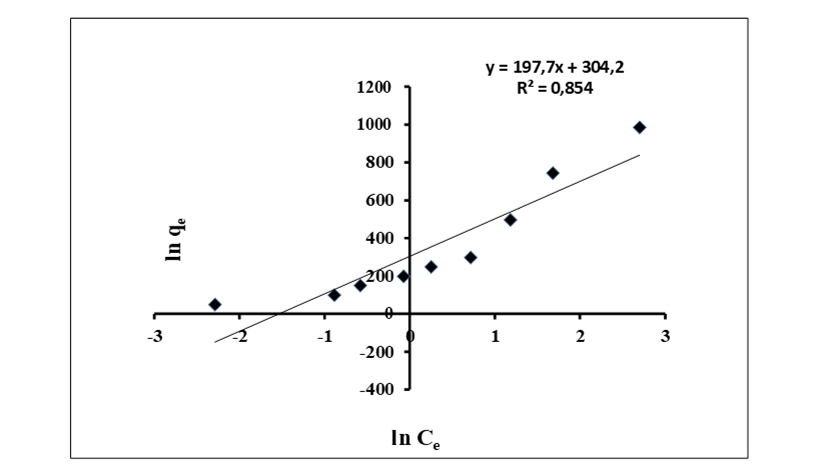
**

**a**

**
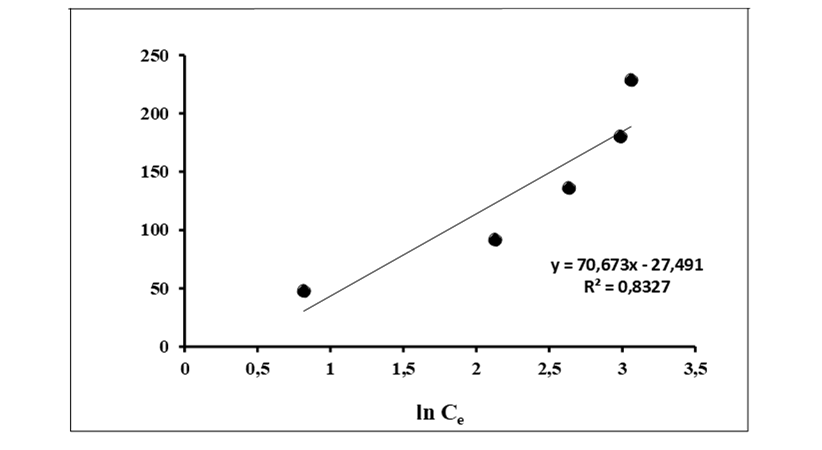
**

**b**

**Supplementary Figure S4.** Temkin adsorption plot for a) RB 19, b) RR195 adsorption.


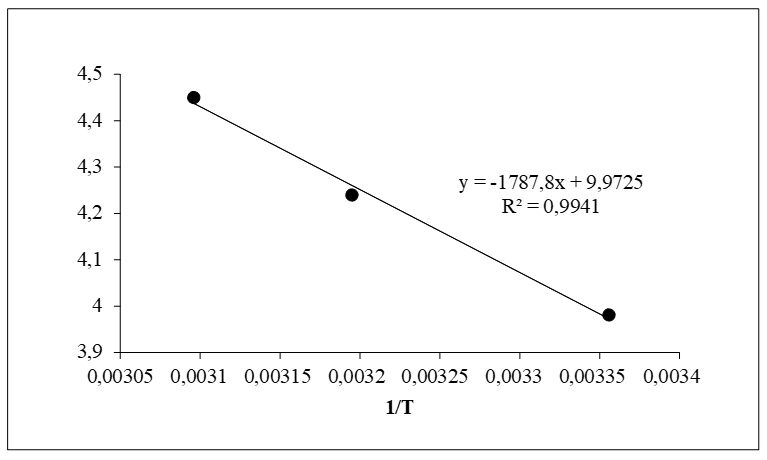


**a**


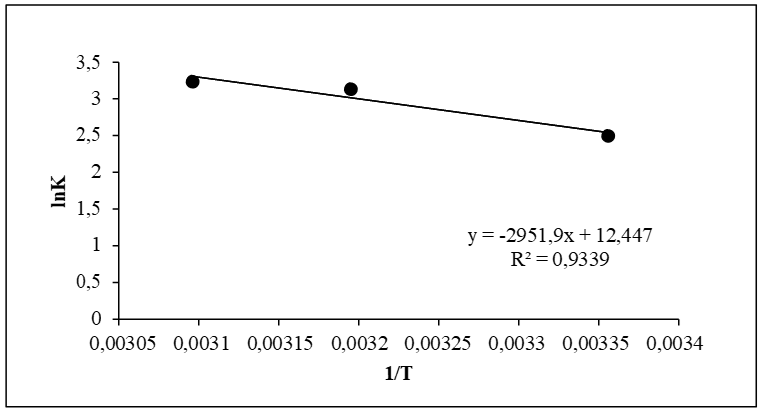


**B**

**Supplementary Figure S5.** Van’t Hoff plot of a) RB19, b) RR195 adsorption.
